# 1589. Real-World Outcomes with Cabotegravir/Rilpivirine: Does Duration of Pre-treatment Viral Suppression Matter?

**DOI:** 10.1093/ofid/ofad500.1424

**Published:** 2023-11-27

**Authors:** Daniel Mesa, Pandit Neha, Patrick Ryscavage, Sarah Schmalzle

**Affiliations:** University of Maryland School of Pharmacy, Baltimore, Maryland; University of Maryland School of Pharmacy, Baltimore, Maryland; University of Maryland, Baltimore, Baltimore, MD; University of Maryland School of Medicine, Baltimore, Maryland

## Abstract

**Background:**

The efficacy and safety of long-acting injectable, cabotegravir/rilpivirine (CAB/RPV), was established in people with HIV (PHIV) on a stable oral antiretroviral (ART) regimen with viral suppression ≥ 6 months prior to CAB/RPV initiation and with no history of treatment failure. Given the design of the CAB/RPV clinical studies, there have been varying recommendations for its initiation in real-world settings due to the lack of data in patients with either incomplete, or shorter duration of pre-treatment viral suppression. The objective of this study was to describe virologic outcomes among patients on CAB/RPV based on duration of pre-treatment viral suppression.

**Methods:**

This was a single-center, retrospective, observational study of PHIV initiated on CAB/RPV at an HIV clinic in Baltimore, MD, between Jan 2021-Dec 2022. Patients were stratified by duration of viral suppression prior to CAB/RPV initiation. Viral suppression was evaluated at weeks 8-24 post CAB/RPV initiation and stratified by body mass index (BMI), baseline drug resistance, and CAB/RPV dosing frequency.

**Results:**

A total of 78 patients were included in this study. The mean age was 47 years, 41% were female at birth, and 92% were Black/African American. At weeks 8-24, 62 patients had HIV RNAs available with 87% (54/62) having achieved viral suppression (< 50 copies/ml). Viral suppression was achieved in 94% (n=33/35), 67% (2/3), and 78% (18/23) of patients who had been virologically suppressed for > 12 months, 6-12 months, and 0-6 months, respectively. Of the 8 patients that did not achieve viral suppression, 6 were able to suppress on CAB/RPV between 125-364 days, 1 had no subsequent HIV RNA result available, and 1 discontinued treatment due to increase in HIV RNA but had been durably suppressed for over 12 months prior to CAB/RPV initiation. We found no differences between viral suppression on CAB/RPV and BMI, baseline mutations, and dosing frequency.

**Conclusion:**

Virologic outcomes did not differ based on duration of viral suppression prior to initiation of CAB/RPV. Though larger studies should be completed, long-acting CAB/RPV has remained effective in patients with variable durations of pre-treatment viral suppression and in patients who did not have viral suppression prior to switch.

**Disclosures:**

**All Authors**: No reported disclosures

